# The Starvation of Prisoners

**Published:** 1865-06

**Authors:** 


### THE STARVATION OF PRISONERS.

If this last of modern wars, now so happily ended, has shown how great has been the advance towards perfection in the art of warfare by an enlightened and inventive people, it has none the less clearly demonstrated how stationary remains man's capabilities for evil in all time. Moved by disappointed ambition, hatred, and revenge, cultivated men of the nineteenth century have proved to be as thoroughly brutal and unchristian as the lowest savages in Africa. The murder of our gentle President was the last of a series of the most inhuman acts that human beings ever devised, but it was far from being the worst of them. In the midst of the judicial investigation which appears to show beyond question the complicity of authenticated and important rebel agents, if not of the chief himself, in this long-planned assassination, the details of a still fouler plot are brought to us. Failing in their designs to burn the inhabitants of our prosperous cities in their beds, the rebel emissaries conceived the monstrous plan of calling the pestilence to their aid, and one of our own profession among them, if it can be believed, was found vile enough to go to its haunt and procure the means of spreading it among the crowded capitals of the North. The Bermuda authorities can scarcely be charged with any exaggeration or over-friendliness to us in their account of this matter, for the accomplice in this fiendish attempt was committed to jail in default of bail of only £50.

But far exceeding any of these crimes in malignancy and depravity, is the process of maiming or slow death by starvation and exposure, which has been systematically practised upon the thousands of our poor fellows in Southern prisons. Concerning the acts and the guilty agents in this instance, there can be no possible doubt; the rebel authorities, civil and military, are personally responsible for this most cruel and wholesale murder. The horrors of Belle Isle, and of the slaughter-pen at Andersonville, where many thousands of our soldiers were slowly starved to death, have been published to the world by the Sanitary Commission, but scarcely any notice has been taken of these barbarities abroad. Some of the latest English journals, in the very numbers in which they express the utmost horror of the last assassination, deny the truth of these statements altogether, and assert that an act of charity on the part of the rebel government, in setting free a few sick prisoners, that they might see their homes once more, has been basely perverted in this manner by the Northern press. Fortunately, we are able to support these statements, perfectly accurate in every particular, by other testimony of a character which English medical journals, at least, will not dare to question. In March, of the present year, 9000 returned prisoners were received within our lines at Wilmington, N. C., of whom 2500 were in such a state that they could not be moved farther North. Dr. J. C. Dalton, Professor of Physiology and Microscopic Anatomy, in the College of Physicians and Surgeons, New York, and author of the widely-known treatise on Human Physiology, and Dr. C. R. Agnew, of the Sanitary Commission, were selected to visit them and examine into their condition, for the purpose of determining whether they were suffering from disease, or in consequence of starvation and exposure. The latter gentleman in his report states:—

"I had several interviews with citizens of Wilmington who had seen our prisoners as they were brought into the city for exchange, with a view of ascertaining what their impressions were, as to the motives which influenced the rebel officers in the management of squads in their respective commands. I found that some of the rebel officers in charge of our returned prisoners had permitted the citizens to furnish them food, while others had forbidden all access to the pens in which the men were quartered; and one, a rebel captain, having charge of about a thousand men, had gone with his drawn sword and knocked the food from the hands of the famished men, informing the citizens who had furnished it, 'that the best thing that could happen to the Yankees was to be starved, and thus expiate the crime which they had committed in invading Southern territory, and destroying the peace of Southern homes.'"

Dr. Dalton spent two days in visiting the hospitals containing the returned prisoners, and says:—

"But the greatest and most pitiful necessities were among our returned prisoners. No description can do justice to their miserable condition, because nothing but an actual inspection of them, in considerable numbers, can show that the wretched faces and figures that present themselves everywhere, are not the isolated and exceptional effects of severe illness, but the general result of a uniform and long-continued process of starvation and misery. There were degrees, of course, in which this condition was more or less marked. The better cases were walking about the streets, perhaps barefooted, or with no other clothing than a pair of white cotton drawers, and an old blanket or overcoat, both equally ragged. In these, the slow, dragging gait, listless manner, and cavernous, inexpressive look of the face, together with the general emaciation, formed a peculiar aspect by which they alone attracted the attention of the passer-by, and by which they were at once distinguished from the other convalescent soldiers. There was no occasion to inquire in Wilmington which were our returned prisoners; after half a day's experience, any one could distinguish them at a glance. Many of them, who had strength to crawl about in this manner, were prevented from doing so by the want of clothing. Major Randlete, the Provost Marshal of Wilmington, told me that on one day forty of these men came into our lines *absolutely as naked as they were born.* I inquired of a considerable number of them, whom I saw in the hospitals confined to their beds—naked, or with only a shirt, and covered with a hospital blanket—what had become of their clothing, and was told that they had thrown away what remained as soon as they could obtain shelter, because it was so ragged, filthy, and full of vermin. One of them, on being told that the Sanitary Commission had sent them flannel shirts and drawers, caught at the word with a childish eagerness, and repeated the good news to his companions with a faint, half-imbecile smile, as long as I was within hearing. With the great majority of the feebler ones, personal cleanliness was a thing which they appeared to have forgotten. They no longer retained sufficient strength, either of mind or body, to appreciate or correct the degredation to which months of unavoidable uncleanliness had reduced them. In the most extreme cases, the condition of the mind, as well as the expression of the face, was absolutely *fatuous*, and the aspect of the patient was not that of a strong man reduced by illness, but that of an idiotic pauper, who had been such from his birth. Nevertheless, several of the surgeons informed me that the condition of the patients had visibly improved since their reception, and that I could not then form an adequate idea of what it was when they entered our lines. In that case it must have been lamentable beyond description.

"The testimony of both men and officers was uniform as to the causes of their unnatural condition. These causes were—1st, starvation; and 2d, exposure. Only such officers and men as could procure money were able to obtain anything like sufficient nourishment. Some of them told me that during the entire winter they had received absolutely no meat; a pint of cornmeal, often with the cob ground in, sometimes with and sometimes without salt, a handful of 'cow peas,' and sometimes sorghum molasses, constituted their usual ration. When in hospital, they had only very thin cornmeal gruel, and a little corn-bread. To the debility occasioned by this insufficient food, was added that resulting from exposure. It was a common thing for a prisoner, immediately on being taken, to be stripped of his clothing—shoes, socks, pantaloons, shirt, and drawers—and to be left with only an old and worn-out pair of drawers, and, perhaps, an equally worn-out shirt and blanket given him in exchange This robbery of clothing was also practised, more or less, upon officers. Even an assistant-surgeon who was captured within four miles of Richmond, told me he was robbed of his flannel shirt, while standing in front of the Libby Prison, and in presence of the rebel officer in charge of the squad. This was immediately after his arrival in the city, and when he had been for the three days succeeding his capture, entirely without food. With the scanty clothing thus left them, the men were kept during the winter, often without any shelter, excepting such as they could contrive to provide by excavating a sort of rifle-pit in the ground, and covering it with old blankets or canvas, as their supply of fuel was insufficient, and sometimes entirely wanting; even in the hospitals, their suffering from cold was very great.

"One of the most melancholy sights in Wilmington, was that to be seen at the 'Geer' Hospitals. In these hospitals were collected all those patients who had lost their feet, either wholly or in part, by freezing, from their exposure during the past winter, and this in a well-wooded country. In some of them, two or three toes only, on one or both feet, were gangrened, and in process of separating by ulceration; in others, both feet had entirely separated, and the patients were awaiting the time when their general strength, and the condition of the stump, would warrant a final amputation. In many cases, the patients ascribed this gangrene directly to frostbites received on particular occasions; in others, to their illness from which they were suffering—generally fever combined with exposure. My own impression, derived from the result of many inquiries, was that it was generally due to a continuous depression of the vital energies from starvation and neglect, resulting gradually in a destruction of the life of those parts most exposed to the cold and the weather."

The accompanying extract from a letter written by Dr. Agnew, dated Wilmington, March 20th, 1865, paints in such terrible colors the appearance of these poor victims of rebel hate, that we have no word to add to their appeal to the civilized world for judgment on those who permitted and those who did such deeds.

"General Abbott, who received our poor fellows in the exchange, has just told me that language would utterly fail to describe their condition. Filth, rags, nakedness, starvation, were personified. Many of the men were in a state of mind resembling idiocy, unable to tell their names, and lost to all sense of modesty, unconscious of their nakedness and personal condition. Some of them moved about on their hands and knees, unable to stand upon their gangrenous feet, looking up like hungry dogs, beseeching the observer for a bite of bread or a sup of water. Some of them hitched along on their hands and buttocks, pushing gangrenous feet, literally reduced to bone and shreds, before them. Others leaned upon staves, and glared from sunken eyes through the parchment-like slits of their open eyelids into space, without having the power to fix an intelligent gaze upon passing objects. Others giggled, and smirked, and hobbled, like starved idiots; while some adamantine figures walked erect, as though they meant to move the skeleton homewards so long as vitality enough remained to enable them to do so. To see the men who remain here in hospital, would move a heart as hard and cold as marble. Their condition is that of men who have for months suffered chronic starvation. Their arms and legs look like coarse reeds with bulbous joints. Their faces look as though a skilful taxidermist had drawn tanned skin over the bare skull, and then placed false eyes in the orbital cavities. They defy description. It would take a pen expert in the use of every term known to the anatomist and the physician, to begin to expose their fearful condition."—*Boston Medical and Surgical Journal.*

